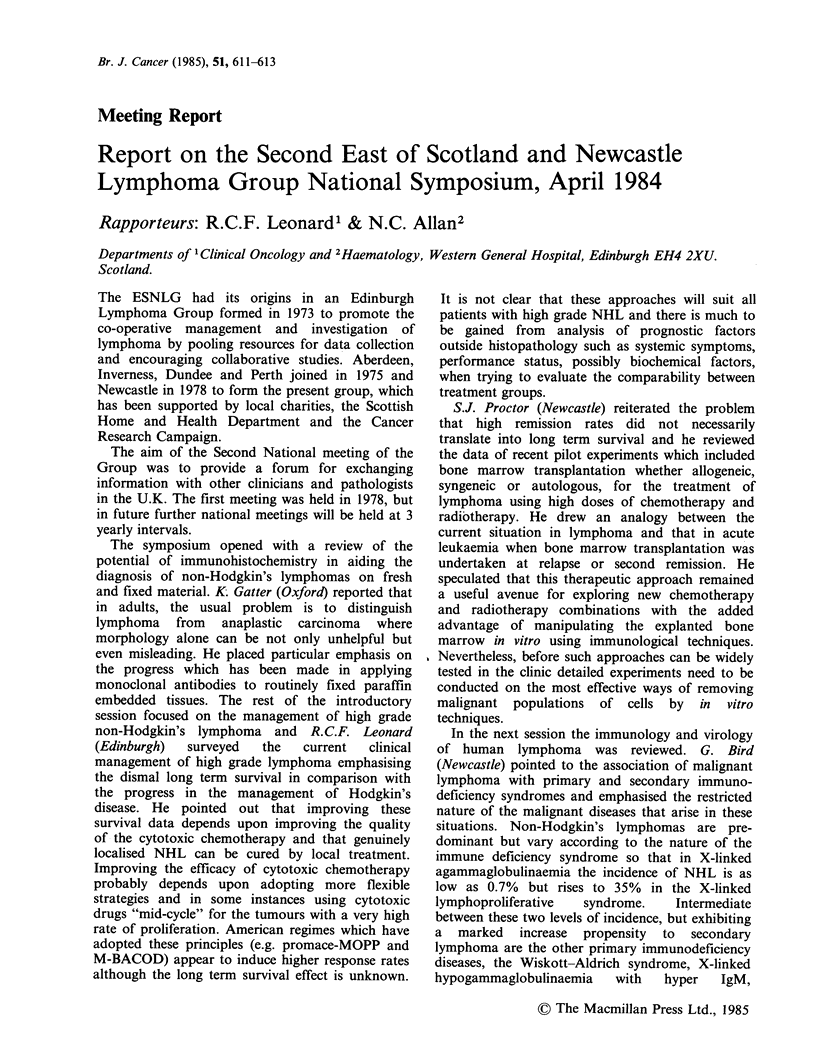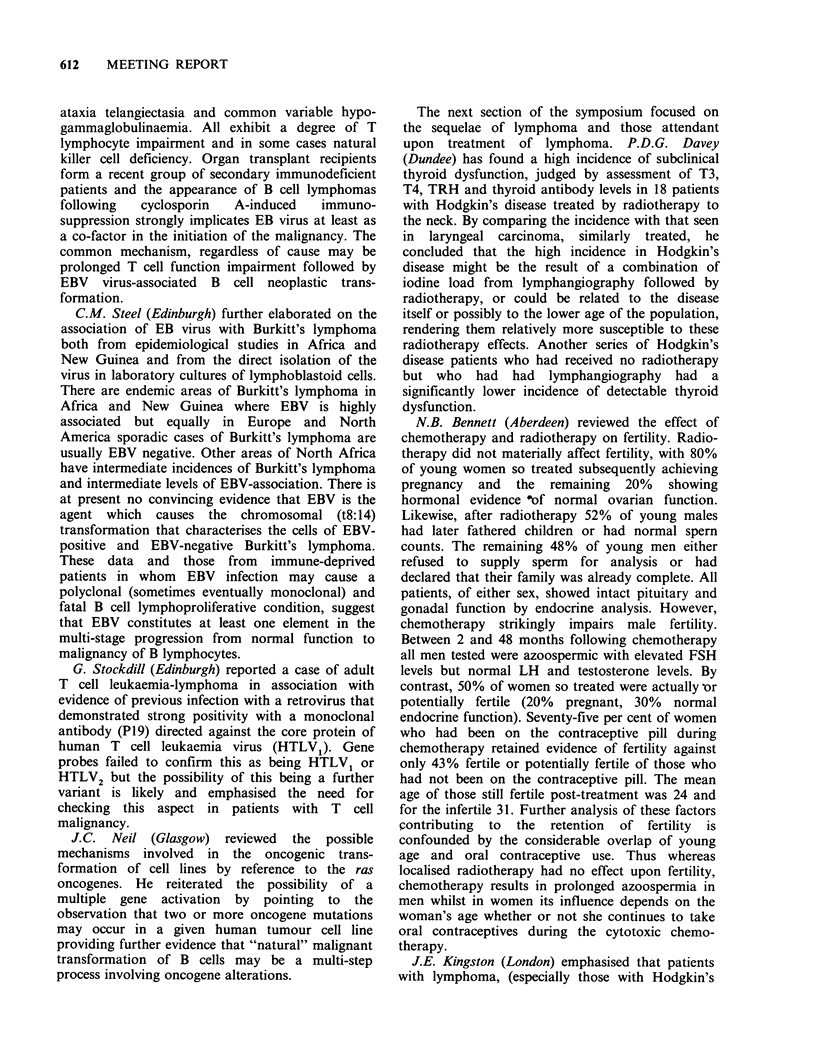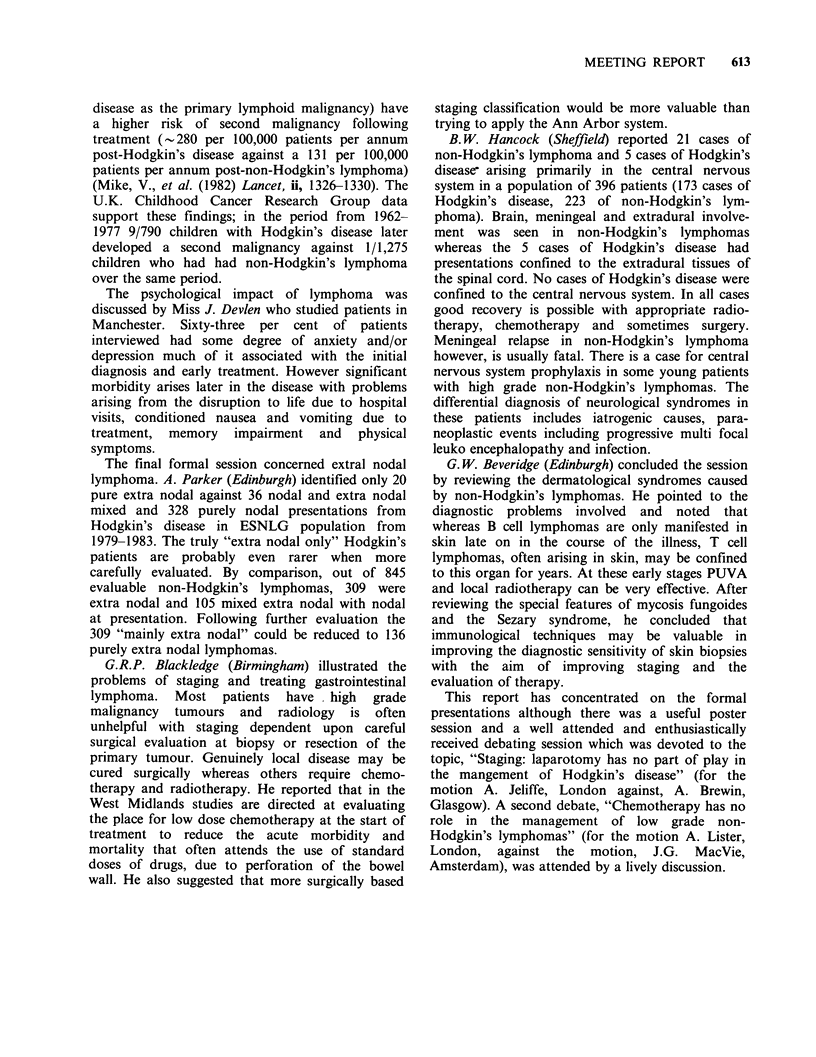# Report on the Second East of Scotland and Newcastle Lymphoma Group National Symposium, April 1984

**Published:** 1985-04

**Authors:** 


*Br. J. Cancer* (1985), **51**, 611–613

### Meeting Report

### Report on the Second East of Scotland and Newcastle Lymphoma Group National Symposium, April 1984

*Rapporteurs*: R.C.F. Leonard[1] & N.C. Allan[2]

*Departments of* [1]*Clinical Oncology and* [2]*Haematology, Western General Hospital, Edinburgh EH4 2XU. Scotland.*

The ESNLG had its origins in an Edinburgh Lymphoma Group formed in 1973 to promote the co-operative management and investigation of lymphoma by pooling resources for data collection and encouraging collaborative studies. Aberdeen, Inverness, Dundee and Perth joined in 1975 and Newcastle in 1978 to form the present group, which has been supported by local charities, the Scottish Home and Health Department and the Cancer Research Campaign.

The aim of the Second National meeting of the Group was to provide a forum for exchanging information with other clinicians and pathologists in the U.K. The first meeting was held in 1978, but in future further national meetings will be held at 3 yearly intervals.

The symposium opened with a review of the potential of immunohistochemistry in aiding the diagnosis of non-Hodgkin's lymphomas on fresh and fixed material. *K. Gatter* (*Oxford*) reported that in adults, the usual problem is to distinguish lymphoma from anaplastic carcinoma where morphology alone can be not only unhelpful but even misleading. He placed particular emphasis on the progress which has been made in applying monoclonal antibodies to routinely fixed paraffin embedded tissues. The rest of the introductory session focused on the management of high grade non-Hodgkin's lymphoma and *R.C.F. Leonard* (*Edinburgh*) surveyed the current clinical management of high grade lymphoma emphasising the dismal long term survival in comparison with the progress in the management of Hodgkin's disease. He pointed out that improving these survival data depends upon improving the quality of the cytotoxic chemotherapy and that genuinely localised NHL can be cured by local treatment. Improving the efficacy of cytotoxic chemotherapy probably depends upon adopting more flexible strategies and in some instances using cytotoxic drugs "mid-cycle" for the tumours with a very high rate of proliferation. American regimes which have adopted these principles (e.g. promace-MOPP and M-BACOD) appear to induce higher response rates although the long term survival effect is unknown. It is not clear that these approaches will suit all patients with high grade NHL and there is much to be gained from analysis of prognostic factors outside histopathology such as systemic symptoms, performance status, possibly biochemical factors, when trying to evaluate the comparability between treatment groups.

*S.J. Proctor* (*Newcastle*) reiterated the problem that high remission rates did not necessarily translate into long term survival and he reviewed the data of recent pilot experiments which included bone marrow transplantation whether allogeneic, syngeneic or autologous, for the treatment of lymphoma using high doses of chemotherapy and radiotherapy. He drew an analogy between the current situation in lymphoma and that in acute leukaemia when bone marrow transplantation was undertaken at relapse or second remission. He speculated that this therapeutic approach remained a useful avenue for exploring new chemotherapy and radiotherapy combinations with the added advantage of manipulating the explanted bone marrow *in vitro* using immunological techniques. Nevertheless, before such approaches can be widely tested in the clinic detailed experiments need to be conducted on the most effective ways of removing malignant populations of cells by *in vitro* techniques.

In the next session the immunology and virology of human lymphoma was reviewed. *G. Bird* (*Newcastle*) pointed to the association of malignant lymphoma with primary and secondary immunodeficiency syndromes and emphasised the restricted nature of the malignant diseases that arise in these situations. Non-Hodgkin's lymphomas are predominant but vary according to the nature of the immune deficiency syndrome so that in X-linked agammaglobulinaemia the incidence of NHL is as low as 0.7% but rises to 35% in the X-linked lymphoproliferative syndrome. Intermediate between these two levels of incidence, but exhibiting a marked increase propensity to secondary lymphoma are the other primary immunodeficiency diseases, the Wiskott–Aldrich syndrome, X-linked hypogammaglobulinaemia with hyper IgM,

© The Macmillan Press Ltd., 1985

ataxia telangiectasia and common variable hypogammaglobulinaemia. All exhibit a degree of T lymphocyte impairment and in some cases natural killer cell deficiency. Organ transplant recipients form a recent group of secondary immunodeficient patients and the appearance of B cell lymphomas following cyclosporin A-induced immunosuppression strongly implicates EB virus at least as a co-factor in the initiation of the malignancy. The common mechanism, regardless of cause may be prolonged T cell function impairment followed by EBV virus-associated B cell neoplastic transformation.

*C.M. Steel* (*Edinburgh*) further elaborated on the association of EB virus with Burkitt's lymphoma both from epidemiological studies in Africa and New Guinea and from the direct isolation of the virus in laboratory cultures of lymphoblastoid cells. There are endemic areas of Burkitt's lymphoma in Africa and New Guinea where EBV is highly associated but equally in Europe and North America sporadic cases of Burkitt's lymphoma are usually EBV negative. Other areas of North Africa have intermediate incidences of Burkitt's lymphoma and intermediate levels of EBV-association. There is at present no convincing evidence that EBV is the agent which causes the chromosomal (t8:14) transformation that characterises the cells of EBV-positive and EBV-negative Burkitt's lymphoma. These data and those from immune-deprived patients in whom EBV infection may cause a polyclonal (sometimes eventually monoclonal) and fatal B cell lymphoproliferative condition, suggest that EBV constitutes at least one element in the multi-stage progression from normal function to malignancy of B lymphocytes.

*G. Stockdill* (*Edinburgh*) reported a case of adult T cell leukaemia-lymphoma in association with evidence of previous infection with a retrovirus that demonstrated strong positivity with a monoclonal antibody (P19) directed against the core protein of human T cell leukaemia virus ($HTLV_1$). Gene probes failed to confirm this as being $HTLV_1$ or $HTLV_2$ but the possibility of this being a further variant is likely and emphasised the need for checking this aspect in patients with T cell malignancy.

*J.C. Neil* (*Glasgow*) reviewed the possible mechanisms involved in the oncogenic transformation of cell lines by reference to the *ras* oncogenes. He reiterated the possibility of a multiple gene activation by pointing to the observation that two or more oncogene mutations may occur in a given human tumour cell line providing further evidence that "natural" malignant transformation of B cells may be a multi-step process involving oncogene alterations.

The next section of the symposium focused on the sequelae of lymphoma and those attendant upon treatment of lymphoma. *P.D.G. Davey* (*Dundee*) has found a high incidence of subclinical thyroid dysfunction, judged by assessment of T3, T4, TRH and thyroid antibody levels in 18 patients with Hodgkin's disease treated by radiotherapy to the neck. By comparing the incidence with that seen in laryngeal carcinoma, similarly treated, he concluded that the high incidence in Hodgkin's disease might be the result of a combination of iodine load from lymphangiography followed by radiotherapy, or could be related to the disease itself or possibly to the lower age of the population, rendering them relatively more susceptible to these radiotherapy effects. Another series of Hodgkin's disease patients who had received no radiotherapy but who had had lymphangiography had a significantly lower incidence of detectable thyroid dysfunction.

*N.B. Bennett* (*Aberdeen*) reviewed the effect of chemotherapy and radiotherapy on fertility. Radiotherapy did not materially affect fertility, with 80% of young women so treated subsequently achieving pregnancy and the remaining 20% showing hormonal evidence of normal ovarian function. Likewise, after radiotherapy 52% of young males had later fathered children or had normal spern counts. The remaining 48% of young men either refused to supply sperm for analysis or had declared that their family was already complete. All patients, of either sex, showed intact pituitary and gonadal function by endocrine analysis. However, chemotherapy strikingly impairs male fertility. Between 2 and 48 months following chemotherapy all men tested were azoospermic with elevated FSH levels but normal LH and testosterone levels. By contrast, 50% of women so treated were actually or potentially fertile (20% pregnant, 30% normal endocrine function). Seventy-five per cent of women who had been on the contraceptive pill during chemotherapy retained evidence of fertility against only 43% fertile or potentially fertile of those who had not been on the contraceptive pill. The mean age of those still fertile post-treatment was 24 and for the infertile 31. Further analysis of these factors contributing to the retention of fertility is confounded by the considerable overlap of young age and oral contraceptive use. Thus whereas localised radiotherapy had no effect upon fertility, chemotherapy results in prolonged azoospermia in men whilst in women its influence depends on the woman's age whether or not she continues to take oral contraceptives during the cytotoxic chemotherapy.

*J.E. Kingston* (*London*) emphasised that patients with lymphoma, (especially those with Hodgkin's disease as the primary lymphoid malignancy) have a higher risk of second malignancy following treatment (~280 per 100,000 patients per annum post-Hodgkin's disease against a 131 per 100,000 patients per annum post-non-Hodgkin's lymphoma) (Mike, V., *et al.* (1982) *Lancet*, **ii**, 1326–1330). The U.K. Childhood Cancer Research Group data support these findings; in the period from 1962–1977 9/790 children with Hodgkin's disease later developed a second malignancy against 1/1,275 children who had had non-Hodgkin's lymphoma over the same period.

The psychological impact of lymphoma was discussed by Miss *J. Devlen* who studied patients in Manchester. Sixty-three per cent of patients interviewed had some degree of anxiety and/or depression much of it associated with the initial diagnosis and early treatment. However significant morbidity arises later in the disease with problems arising from the disruption to life due to hospital visits, conditioned nausea and vomiting due to treatment, memory impairment and physical symptoms.

The final formal session concerned extral nodal lymphoma. *A. Parker* (*Edinburgh*) identified only 20 pure extra nodal against 36 nodal and extra nodal mixed and 328 purely nodal presentations from Hodgkin's disease in ESNLG population from 1979–1983. The truly "extra nodal only" Hodgkin's patients are probably even rarer when more carefully evaluated. By comparison, out of 845 evaluable non-Hodgkin's lymphomas, 309 were extra nodal and 105 mixed extra nodal with nodal at presentation. Following further evaluation the 309 "mainly extra nodal" could be reduced to 136 purely extra nodal lymphomas.

*G.R.P. Blackledge* (*Birmingham*) illustrated the problems of staging and treating gastrointestinal lymphoma. Most patients have high grade malignancy tumours and radiology is often unhelpful with staging dependent upon careful surgical evaluation at biopsy or resection of the primary tumour. Genuinely local disease may be cured surgically whereas others require chemotherapy and radiotherapy. He reported that in the West Midlands studies are directed at evaluating the place for low dose chemotherapy at the start of treatment to reduce the acute morbidity and mortality that often attends the use of standard doses of drugs, due to perforation of the bowel wall. He also suggested that more surgically based staging classification would be more valuable than trying to apply the Ann Arbor system.

*B.W. Hancock* (*Sheffield*) reported 21 cases of non-Hodgkin's lymphoma and 5 cases of Hodgkin's disease arising primarily in the central nervous system in a population of 396 patients (173 cases of Hodgkin's disease, 223 of non-Hodgkin's lymphoma). Brain, meningeal and extradural involvement was seen in non-Hodgkin's lymphomas whereas the 5 cases of Hodgkin's disease had presentations confined to the extradural tissues of the spinal cord. No cases of Hodgkin's disease were confined to the central nervous system. In all cases good recovery is possible with appropriate radiotherapy, chemotherapy and sometimes surgery. Meningeal relapse in non-Hodgkin's lymphoma however, is usually fatal. There is a case for central nervous system prophylaxis in some young patients with high grade non-Hodgkin's lymphomas. The differential diagnosis of neurological syndromes in these patients includes iatrogenic causes, paraneoplastic events including progressive multi focal leuko encephalopathy and infection.

*G.W. Beveridge* (*Edinburgh*) concluded the session by reviewing the dermatological syndromes caused by non-Hodgkin's lymphomas. He pointed to the diagnostic problems involved and noted that whereas B cell lymphomas are only manifested in skin late on in the course of the illness, T cell lymphomas, often arising in skin, may be confined to this organ for years. At these early stages PUVA and local radiotherapy can be very effective. After reviewing the special features of mycosis fungoides and the Sezary syndrome, he concluded that immunological techniques may be valuable in improving the diagnostic sensitivity of skin biopsies with the aim of improving staging and the evaluation of therapy.

This report has concentrated on the formal presentations although there was a useful poster session and a well attended and enthusiastically received debating session which was devoted to the topic, "Staging: laparotomy has no part of play in the mangement of Hodgkin's disease" (for the motion A. Jeliffe, London against, A. Brewin, Glasgow). A second debate, "Chemotherapy has no role in the management of low grade non-Hodgkin's lymphomas" (for the motion A. Lister, London, against the motion, J.G. MacVie, Amsterdam), was attended by a lively discussion.